# Complete Genome Sequence Analysis of Echovirus 24 Associated with Hand-Foot-and-Mouth Disease in China in 2012

**DOI:** 10.1128/genomeA.01456-14

**Published:** 2015-02-05

**Authors:** Lian-lian Bian, Xin Yao, Qun-ying Mao, Fan Gao, Yi-ping Wang, Qiang Ye, Feng-cai Zhu, Zheng-lun Liang

**Affiliations:** a National Institutes for Food and Drug Control, Beijing, China; b Jiangsu Provincial Center for Disease Control and Prevention, Nanjing, China

## Abstract

Echovirus 24 belongs to human enterovirus B species in the family *Picornaviridae*. Here, we report the whole-genome sequences of a novel complete genome sequence of a recombinant (echovirus 24) Echo 24 strain, PZ18/JS/2012, which was isolated from a patient with hand-foot-and-mouth disease in China.

## GENOME ANNOUNCEMENT

Echovirus 24 (Echo 24) belongs to the genus *Enterovirus*, family *Picornaviridae*, and is one of 43 serotypes of the human enterovirus B group (1). Echo 24 can cause severe neurological diseases, such as acute flaccid paralysis (AFP), encephalitis, and aseptic meningitis (2–7). In recent decades, Echo 24-associated neurological diseases have been documented in Asia (2), Africa (3, 4), the United States (5) and mainland China (6, 7), while there has been no reported case of Eco24 infection-associated hand-foot-and-mouth Disease (HFMD) in the world. To date, only one complete genome sequence of Echo 24 is available in GenBank, prototype strain DeCamp (USA, 1956, GenBank accession no. AY302548) (8).

Here, we report the complete genome of an Echo 24 strain isolated from a 9-month-old boy clinically diagnosed in 2012 with HFMD in Jiangsu province of China, who had rashes and herpes on his hand, foot, oral cavity, and cheek. Throat and rectal swabs were collected within 24 h after the onset of the rashes and herpes. After serial passage in an RD cell line, the recovered isolate was named PZ18/JS/2012. Briefly, viral RNA was extracted from viral culture with an RNeasy minikit (Qiagen), and cDNA was produced by using reverse transcriptase (Invitrogen). Four pairs of primers were used to generate the amplicons spanning the entire viral genome. All sequencing was carried out using an ABI 3730 Sanger-based genetic analyzer, and the sequences were assembled using DNAStar Lasergene 7.0. Sequence alignment was performed using MegAlign. Phylogenetic trees were constructed using the MEGA 5.0 software. Similarity plot analyses were performed using SimPlot 3.5.1.

The complete genome of PZ18/JS/2012 consists of 7,373 nucleotides (nt), including a 746-nt 5′ untranslated region (5′-UTR), a 36-nt 3′ untranslated region (3′-UTR), and a 6,591-nt open reading frame (ORF) that encodes a 2,197-amino-acid polyprotein. The genome organization of this virus is identical to that of DeCamp. Phylogenetic analysis based on the complete genomes of DeCamp and PZ18/JS/2012 showed that the nucleotide identity is 80.4%, and the nucleotide identities of different genome regions are 72.7% to 82.2%. The results of the phylogenetic analysis conducted using the neighbor-joining method showed that the VP1 sequences of Echo 24 in GenBank are clustered into three branches, A, B, and C. DeCamp belongs to cluster A, which shows 74.6 to ~82.3% nucleotide similarity compared with clusters B and C. Cluster B is composed of 3 sublineages (B1, B2, and B3). The sequences from isolates from Bangladesh and India are all clustered in sublineage B1, while the majority of the Chinese isolates are in sublineages B2 and B3. Interestingly, PZ18/JS/2012 is clustered in lineage C with the sequence of 96-AFP/YN/CHN/2010 (GenBank accession no. AB740165) isolated from an AFP patient in Yunnan, and this isolate showed the highest homology (93.6%) with PZ18/JS/2012. A similarity analysis using SimPlot 3.5.1 indicated that the PZ18/JS/2012 strain recombined with CB1/SD/CHN/2011 (GenBank accession no. JX976769) at the 2C region (general location, position 4870). This is the first evidence for natural intertypic recombination for Echo 24.

### Nucleotide sequence accession number.

The complete genomic sequence of PZ18/JS/2012 was deposited in GenBank under the accession no. KP036484.
